# Ammonia-oxidizing bacteria and archaea within biofilters of a commercial recirculating marine aquaculture system

**DOI:** 10.1186/s13568-018-0551-1

**Published:** 2018-02-10

**Authors:** Zhitao Huang, Yuli Jiang, Xiefa Song, Eric Hallerman, Lei Peng, Dengpan Dong, Teng Ma, Jieming Zhai, Wensheng Li

**Affiliations:** 1 Department of Fisheries, Ocean University of China, Qingdao, 266003 People’s Republic of China; 2 Department of Fish and Wildlife Conservation, Virginia Polytechnic Institute and State University, Blacksburg, VA 24061 USA; 3 Rizhao Aquaculture Technology Extension Station, Rizhao, 226600 People’s Republic of China; 4 Laizhou Mingbo Aquatic Co., Ltd., Lai Zhou, 261418 People’s Republic of China

**Keywords:** Water quality, Growth, Microbial community, Recirculating aquaculture system, Hybrid grouper *Epinephelus lanceolatus* × *E. fuscoguttatus*, Metabarcoding

## Abstract

While biofilters are widely used to metabolize ammonia and other wastes in marine recirculating aquaculture systems, the ammonia-oxidizing bacterial and archaeal communities have not been characterized across a diversity of production systems. Using a metagenomics approach, we characterized the ammonia-oxidizing microbiological community of biofilters in a commercial recirculating marine aquaculture system producing hybrid grouper (*Epinephelus lanceolatus* × *E. fuscoguttatus*). Cloning and sequencing of the *amoA* gene showed that nitrifying bacteria included *Nitrosomonas europea*, *N. stercoris*, *N. cryotolerans*, *N. eutropha*, *N. estuarii*, eight strains of *N. marina*, and 15 strains not associated with described species. Nitrifying archaea included eight strains of *Nitrosopumilus maritimus*, *N. koreensis*, *N*. *piranensis*, *N. adriaticus*, undescribed congeners, and other undescribed archaea. The species composition of the bacterial and especially the archaeal communities was beyond that yet reported for aquaculture biofilters. While ammonia flux through the respective communities has yet to be estimated, the diverse environmental adaptations of the bacterial and archaeal communities suggest resilience of function under a range of environmental conditions.

## Introduction

Effective biological filtration is critical to fish production in a recirculating aquaculture system (RAS). Classical characterization of biological filtration involves measurement of chemical parameters (e.g., ammonia, nitrite, and nitrate concentrations, biochemical oxygen demand), and culture-based identification of key species within the microbial consortium. Chemolithotrophic ammonia-oxidizing bacteria (AOB) grow only very slowly, however, and are more difficult to detect in the biofilm than the more-common *Nitrosomonas* and *Nitrosococcus* species. With the advent of metagenomic analyses, the latter may also involve molecular genetic characterization of the microbial community of the biofilm in the biofilter. That is, species-specific DNA sequences—most often of the *16S* rRNA gene—may be amplified, sequenced, and screened against a genomic database to identify the microbes in the biofilm (e.g., Schreier et al. 2010; Huang et al. 2016). An enzyme unique to chemolithotrophic ammonium-oxidizing microbes (McTavish et al. 1993; Bergmann and Hooper 1994) is ammonia monooxygenase (*amoA*), which oxidizes ammonia to the intermediate hydroxylamine (Wood 1986); although the enzyme also oxidizes other substrates (Bock et al. 1986; Hooper 1989), *amoA* is a useful target DNA sequence for detecting and identifying ammonium-oxidizing microbes in environmental samples. Polymerase chain reaction (PCR) assays that target the *amoA* gene are sufficiently sensitive and non-specific to detect a broad range of microbes containing the enzyme in environmental samples (Sinigalliano et al. 1995). While studies of ammonia-oxidizing microbial communities of aquaculture biofilters have been executed (Tal et al. 2003; Sugita et al. 2005; Itoi et al. 2006; Foesel et al. 2008; Urakawa et al. 2008; Schreier et al. 2010; Sauder et al. 2011; Keuter 2011; Sakami et al. 2012; Blancheton et al. 2013; Brown et al. 2013; Kruse et al. 2013; Bagchi et al. 2014; Rurangwa et al. 2014; Michaud et al. 2014; Ruan et al. 2015; Gonzalez-Silva et al. 2016; Huang et al. 2016; Lee et al. 2016; Bartelme et al. 2017; Keuter et al. 2017), molecular genetic and bioinformatic tools as well as DNA sequence archives for bacterial characterization have improved recently, especially for ammonia-oxidizing microbes. Further, characterization of a broader range of marine RAS would deepen our insight into microbial community structure and function under a range of culture conditions.

Groupers (subfamily *Epinephelinae*, family *Serranidae*) comprise a diverse group of predatory fishes widely distributed through tropical and subtropical seas, and have become an important aquaculture product. In China alone, captive production of groupers in 2014 totaled 88,130 metric tons (Fishery Bureau 2015). Hybrid tiger (*Epinephelus fuscoguttatus*) × giant (*E. lanceolatu*s) grouper exhibit rapid growth, thick fillet, high collagen content in meat, and strong disease resistance (Ch’ng and Senoo 2008), and has been commercialized globally (Senoo 2010; Wang et al. 2015). Production is carried out in flow-through systems (Wang et al. 2004), highly intensive culture ponds (Li et al. 2013), and more recently in recirculating aquaculture systems (Yang et al. 2016).

We previously characterized and compared the microbial communities of nine biofilters in five commercial marine recirculating marine aquaculture systems by amplifying and sequencing the *16S* ribosomal RNA (rRNA) gene (Huang et al. 2016). Within the nitrifying community of three biofilters at Laizhou Mongbao Aquatic Co., Ltd., our results showed relatively frequent observations of *Nitrosomonas* sp. (1.1–2.9%) and *Nitrospira* sp. (0.5–7.1%). Ammonia being the more toxic nitrogenous waste to fishes, we aimed in this study to focus more closely on the critical ammonia-oxidizing community. Hence, we chose to sequence the ammonia monooxygenase (*amoA*) gene, identifying the respective bacterial and also archaeal hosts by searching our sequence results against archived *amoA* sequences. Our results revealed a diversity of ammonia-oxidizing bacteria and especially archaea not heretofore known in aquaculture biofilters. We discuss the diversity of species and their respective environmental optima with respect to the resilience of biofilm function under fluctuating production and environmental conditions.

## Methods and materials

### System description

**Fig. 1:**
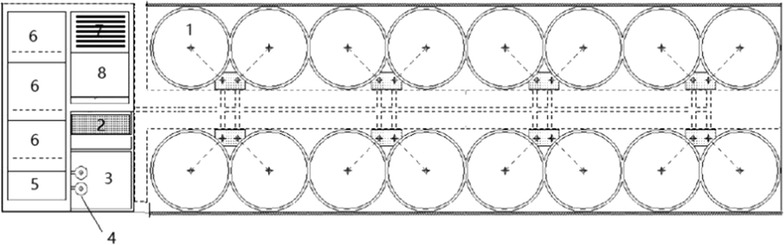
Schematic diagram for RAS producing hybrid grouper (*Epinephelus lanceolatus* × *E. fuscoguttatus*) at Laizhou Mingbo Aquatic Co. The units are: (1) 16 fish rearing tanks, (2) a bowed screen, (3) a sump, (4) two pumps, (5) foam fractionators, (6) series of three biofilters, (7) UV disinfection unit, and (8) dissolved oxygen contact unit

The commercial RAS at Laizhou Mingbo Aquatic Co., Ltd. (Fig. 1) contained 16 fish-rearing tanks that were 7-m inner diameter × 1.0 m-deep. The tanks were filled to depths of 0.8–0.9 m, yielding a water volume in each tank of approximately 33 m^3^. Water was pumped through two 5-cm inner-diameter nozzles above each tank. Water level in each tank was controlled by an outside standpipe connected to the bottom drain. The effluent from the bottom of the tanks first passed through a bowed screen with 0.25-mm screen sieves to remove suspended solids; the filtrate on the screen was removed manually with high-pressure water about 30 min after feeding. The water then passed through a foam fractionator (3 m diameter × 3 m height, 2.5 m water depth) to remove fine solids. A series of three submerged biofilters (3 ml × 3 m W × 5 m H each) containing elastic polyolefin media bio-media (100 m^2^/m^3^) treated ammonia and other metabolic waste products. An ultraviolet light chamber unit with 16 55-W lamps inactivated heterotrophic and coliform bacteria. Micropore diffusers (Tean Technology Co., Ltd., Nanjing, China) injected pure oxygen into the contact unit, and treated water flowed back to the rearing tanks by gravity.

### Fish culture

19,200 hybrid groupers (1200 per tank) were stocked into the RAS for 240 days (April 4th to November 23rd, 2013) for on-growing to harvest size. Initial mean weight was 156.3 ± 11.8 g/fish, mean length was 20.9 ± 1.4 cm, and stocking density was 6.08 kg/m^3^. The fish were hand-fed a commercial diet containing 45% protein and 16% lipid at 1.2–1.5% body weight/day for the first 2 months, and 1% body weight/day thereafter.

Thirty to fifty fish per tank were randomly sampled monthly to determine body weight gain, length gain, specific growth rate, and feed conversion rate. Total ammonia was analyzed weekly using the phenate method and nitrite using the sulfanilamide-NED method (APHA et al. 1995). Temperature and dissolved oxygen were monitored daily using a YSI 85 probe (YSI, Inc., Yellow Springs, OH, USA). pH was measured daily using a YSI pH100m (YSI Inc., Yellow Springs, OH).

### Characterization of biofilter microbial communities

Bio-media from each unit in the series of submerged biofilters were cut into small pieces, and the microbial film was suspended in phosphate-buffered saline solution. After centrifugation at 14,000*g* for 10 min, each supernatant was collected for DNA extraction using the FastDNA Spin Kit for Soil (MP Biomedicals, Illkirch, France) using to the manufacturer’s protocols. The quantity and quality of the extracted DNA were measured using an ND-2000 spectrophotometer (Nanodrop, Inc., Wilmington, DE, USA).

To characterize the ammonia-oxidizing bacterial and archaeal communities, DNA samples were amplified, cloned, and sequenced. The 491-bp fragments of bacterial *amoA* genes and the 635-bp fragments of archaeal *amoA* genes were amplified using the PCR primers and amplification conditions shown in Table 1. Replicate amplifications were pooled and purified by gel electrophoresis using a NucleoSpin Extract II Kit (Clontech Laboratories, Inc., Mountain View, CA, USA). Purified PCR amplicons were cloned using the pGEM-T Easy vector system (Promega, Fitchburg, WI, USA). About 10 positive clones from each sample (70 total) were selected at random for DNA sequencing, which was carried out at BGI (Shenzhen, China). Sequences for *amoA* genes were binned into operational taxonomic units (OTUs) exhibiting 95% nucleotide sequence identity using MOTHUR (Schloss et al. 2009). Cloned sequences of the *amoA* gene for ammonia-oxidizing bacterial and archaeal genes were identified by searching against DNA sequences archived in GenBank using the NCBI Basic Alignment and Search Tool (http://blast.ncbi.nlm.nih.gov/Blast.cgi).

**Table 1:** Technical details for microbial community characterization

Primers	Target taxa	PCR conditions	References
Arch-*amoA*F Arch-*amoA*R	AOA	94 °C for 2 min; followed by 30–35 cycles for 30 s at 94 °C, 45 s at 53 °C, and 45 s at 72 °C, followed by 10 min of final extension at 72 °C	Francis et al. (2005)
*amoA*-*1*F *amoA*-*2*R	AOB	94 °C for 2 min; followed by 30–35 cycles of 30 s at 94 °C, 45 s at 55 °C, and 45 s at 72 °C, followed by 10 min of final extension at 72 °C	Rotthauwe et al. (1997)

*AOA* ammonia-oxidizing archaea, *AOB* ammonia-oxidizing bacteria

We conducted phylogenetic cluster analysis using the unweighted pair group method with arithmetic averaging algorithm to estimate β-diversity and to visualize microbial community diversity (Lozupone and Knight 2005). Neighbor-joining trees were constructed using Molecular Evolutionary Genetics Analysis v. 5.0 (Tamura et al. 2011). In an attempt to root the AOA tree, we included the *amoA* sequences of *Nitrosarcheum* sp. (GenBank Accession Numbers LN823900.1 and LN823940) in our analysis.

### Archiving of new *amoA* sequences

New *amoA* sequences have been deposited in GenBank under Accession Numbers MF959625 to MF959695.

## Results

### Water quality

Quality parameters for treated water returned to the rearing tanks (Table 2) were all appropriate for the production of grouper (Yang et al. 2016). Mean temperature was 23.3 ± 0.8 °C, suitable for grow-out of hybrid grouper. Through the course of production, the mean dissolved oxygen concentration was 7.2 ± 0.3 mg/l, which is close to saturation at 23 °C. Since nitrification was well established in the series of biofilters before culture commenced, neither total ammonia nitrogen (TAN) nor nitrite accumulated during fish production. The mean TAN concentration entering each tank was 0.219 ± 0.012 mg/l, nitrite 0.022 ± 0.002 mg/l, and chemical oxygen demand 0.96 ± 0.06 mg/l. These results show that this series of biofilters metabolized ammonia effectively, providing a suitable context for considering the conditions under which ammonia-oxidizing bacteria and archaea functioned.

**Table 2:** Production and water quality parameters within the recirculating aquaculture system

Final fish density (kg/m^3^)	52.3
Fish survival rate	94%
Feed conversion ratio	1.2
Specific growth rate (%/days)	1.01
Total ammonia nitrogen (mg/l)	0.219 ± 0.012
Chem. oxygen demand (mg/l)	0.96 ± 0.06
Nitrite (mg/l)	0.022 ± 0.002
Dissolved oxygen (mg/l)	7.2 ± 0.3
pH	7.8 ± 0.2
Temperature (°C)	23.3 ± 0.8

### Fish growth and production

Growth performance of the hybrid grouper (*Epinephelus lanceolat*us♂ × *E. fuscoguttatus*♀) through the 240-day on-growing period met expectations for commercial production (Table 2). Starting at 156.3 ± 11.8 g, the grouper grew quickly to 1324 ± 53.6 g. From a low starting density of 6.08 kg/m^3^, final densities reached as high as 52.3 kg/m^3^. No disease outbreaks occurred, and no chemotherapeutics or antibiotics were used during the production period. Survival through the culture period was around 94%. Through the entire production period, the specific daily growth rate was 0.89% body weight/day and the feed conversion ratio was 1.2. Production of hybrid grouper in the recirculating system exhibited higher density, survival rate and feed conversion than in flow-through and intensive pond systems (Wang et al. 2004; Li et al. 2013), indicating high performance of the biofilter and recirculating system.

### Ammonia-oxidizing bacteria

**Fig. 2:**
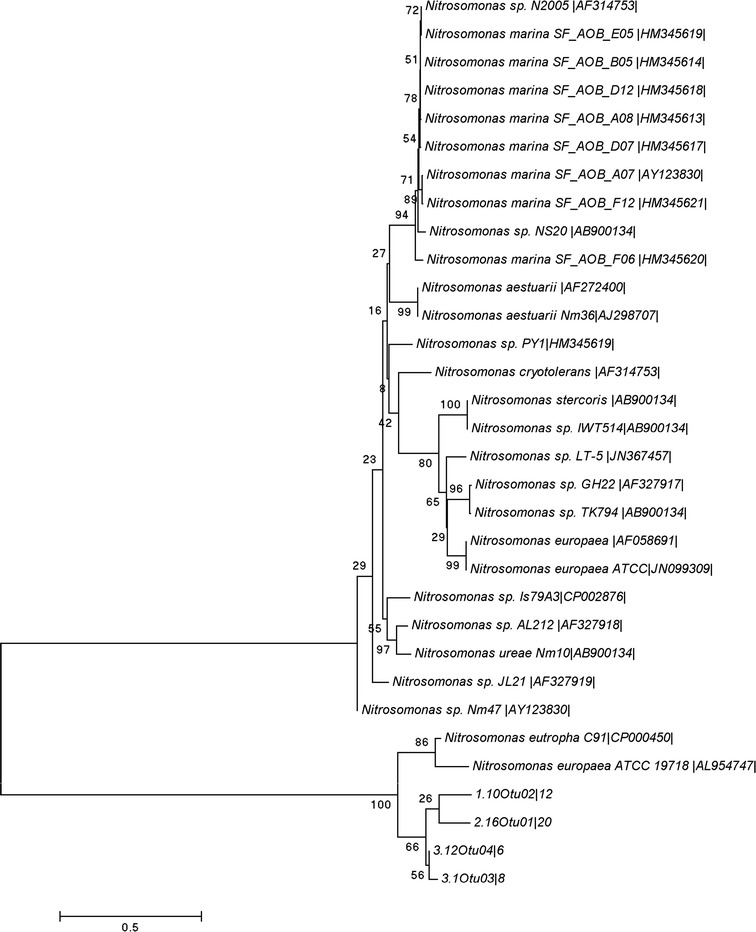
Neighbor-joining tree for DNA sequences of *amoA* genes of ammonia-oxidizing bacteria in recirculating aquaculture systems producing hybrid grouper. Numbers to left of nodes indicate bootstrap support for those nodes. The bar shows phylogenetic distance in numbers of nucleotide substitutions per site

Following amplification of the bacterial *amoA* gene, 46 clones were randomly selected for DNA sequencing, and 31 unique bacterial *amoA* gene sequences were observed. Phylogenetic analysis (Fig. 2) showed that most represented operational taxonomic units (OTUs) within the genus *Nitrosomonas* within the *β*-*Proteobacteria*. This included eight strains of *Nitrosomonas marina*, two *N. aestuari*, one *N. chryotolerans*, one *N. stercornis*, three *Nitrosomonas europaea*, one *N. ureae*, and one *N. eutropha*. The sequences for *N. europaea* [AF058691] and ATCC[JN099309] clustered separately from that for *N. europaea* ATCC19718[AL954747]. That DNA sequences of *N. europaea* are arrayed on both sides of the principal node of the phylogram is likely the consequence of there being two differentiated copies of *amoA* in its genome (Sinigalliano et al. 1995). Not all OTUs were associated with described species, an outcome not unusual in DNA metabarcoding studies (Purkhold et al. 2003; Tang et al. 2014). We observed 11 distinct *amoA* sequences from different strains of *Nitrosomonas* that could not be associated with a described species. Our results also included four unidentified OTUs that could not be related to a known species, but because of their high similarity with other *Nitrosomonas* sequences, seem likely to represent lineages within the genus.

### Ammonia-oxidizing archaea

**Fig. 3:**
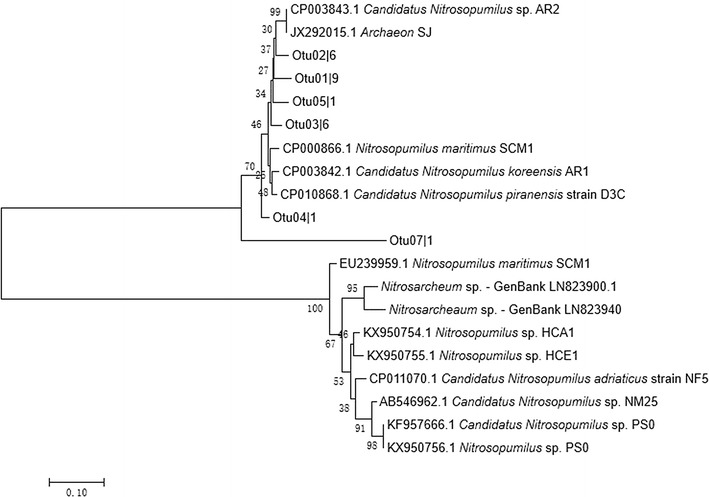
Neighbor-joining tree for DNA sequences of *amoA* genes of ammonia-oxidizing archaea in recirculating aquaculture systems producing hybrid grouper. Numbers to left of nodes indicate bootstrap support for those nodes. The bar shows phylogenetic distance in numbers of nucleotide substitutions per site

From the library for archaeal *amoA* genes, 24 randomly drawn clones were sequenced. Phylogenetic analysis of the 18 unique archaeal *amoA* gene sequences (Fig. 3) showed that they clustered into seven OTUs, and that most of the sequences were homologous to those of *Nitrosopumilus* species. We detected the presence of two different strains of *Nitrosopumilus maritimus* (SCM1 and NAOA6), a common archaeon in seawater; the species having first been isolated from sediment in a marine aquarium (Konneke et al. 2005), its detection in a marine RAS is not surprising. We observed one *amoA* sequence each for *N. koreensis* strain AR1, *N. piranensis* strain D3C, and *N. adriaticus* strain NF5, as well as five strains for unnamed member(s) of the genus, denoted *Nitrosopumilis* sp. AR2, SW, HCA1, NM25, and PS0 in Fig. 3. Our results also include six *amoA* sequences from unidentified archaean OTUs; as noted above, inability to associate environmental DNA sequences with known taxa is not surprising, as metagenomics studies often uncover unknown taxa; this issue is particularly the case for archaea, for which our understanding is still emerging. That DNA sequences for the nominal outgroup, the confamilial *Nitrosarcheum* sp. was grouped within the lower branch for *Nitrosopumilus* species was unexpected, and can be explained by: (1) too little sequence having been analyzed to resolve true phylogenetic relationships, or (2) improperly resolved taxonomy having been incorporated into the archived database.

## Discussion

We characterized the ammonia-oxidizing microbial community of biofilters in a marine recirculating aquaculture system producing hybrid grouper. DNA barcoding using the *amoA* gene showed the AOB community to be dominated by numerous *Nitrosomonas* species and strains and the AOA community by *Nitrosopumilus* species and strains. Our results contrast somewhat with those of earlier studies, at least in part because of methodological advances. Hovanec and DeLong (1996) used the rRNA-based metabarcoding approach and the limited DNA sequence archives of the time, the latter limiting their ability to identify ammonia-oxidizing species in freshwater and saltwater aquaria. Hence, they were limited to reporting that nitrifiers appeared to be dominated by *Nitrosomonas europaea* and its close relatives, while we are able to report 28 species and strains of *Nitrosomonas*. Using denaturing gradient gel electrophoresis (DGGE) and *16S* rRNA sequencing, Tal et al. (2003) detected *N. cryotolerans* and *Nitrospira marina*, as well as heterotrophs (including *Pseudomonas* sp., *Sphingomonas* sp., and *Planctomyces* sp., suggesting the possibility of anaerobic ammonia oxidation) in a system producing gilthead sea bream (*Sparus auratus*). Using a clone library of partial *16S* rRNA gene sequences to characterize the biofilter microbial communities in a marine RAS producing pufferfish *Takifugu rubripes*, Itoi et al. (2006) showed *N. aestuarii*, *Nitrosomonas* sp. Nm143, and two clones forming a separate cluster. Using fluorescent in situ hybridization, Foesel et al. (2008) reported that the most abundant AOB in trickling filters for marine RAS producing gilthead seabream were *Nitrosomonas* sp. Nm143 and *N. marina*. In a review of published studies, Schreier et al. (2010) reported *Nitrosomonas cinnybus*/*nitrosa* and *Nitrosococcus mobilis* in the biofilters of marine RAS. Lee et al. (2016) performed sequencing of *16S* rRNA amplicons of AOB in a recirculating aquaculture system producing sevenband grouper (*Epinephelus fasciatus*) at three temperatures and three salinities. Their results included five *Nitrospira* taxa, four *Nitrosomonas*, two *Nitrospina*, and many unassigned OTUs. Interestingly, the AOB communities they characterized were rather different from ours, even though this being a recent publication, the DNA sequence archive that they used was similarly elaborated. These differences among these communities may be a real consequence of salinity and temperature differences, or may be a consequence of use of *16S* rRNA versus *amoA* barcoding approaches. Characterizing marker lipids and screening *16S* rRNA genes, Kruse et al. (2013) showed that the AOB in moving-bed biofilters of brackish recirculation aquaculture systems producing Pacific white shrimp (*Penaeus vannemei*) and barramundi (*Lates calcarifer*) included four different phylogenetic clusters within the marine sublineage IV of *Nitrospira*. *16S* rRNA barcoding of bacterial communities of biofilters in an RAS producing tongue sole (*Cynoglossus semilaevis*) (Ruan et al. 2015) suggested that the AOB belonged to *Nitrosomonas* clusters; while this result was similar to ours, we were able to show a greater diversity of known and unknown members of the genus. In two RAS producing seabass (*Dicentrarchus labrax*), *16S* rRNA barcoding (Keuter et al. 2017) showed that AOB belonged almost exclusively to *Nitrosomonas*, of which dominant species shifted in both systems over time.

Use of *amoA* metabarcoding and phylogenetic analysis allows greater ability to detect and characterize ammonia-oxidizing microbes, although the state of development of the DNA sequence archive at any point in time constrains the ability to relate amplification products to particular species. Our results builds upon earlier work by making use of better-developed DNA sequence archives and association of more clones with particular species, whose environmental requirements are now better known, as discussed below. Thus, we were able to go farther than Urakawa et al. (2008), many of whose clones could not be associated with species and strain. They identified 5–15 ammonia-oxidizing bacterial species per recirculating aquaculture system, while we identified 31. We observed more species of *Nitrosomonas*, including many strains of *N. marina*. We did not see *Nitrospira* or *Nitrosococcus* species. Urakawa et al. (2008) could characterize clones in Cluster “A” in their archaeal phylogram only as clones related to *N. maritimus*, and the other four clusters only by their derivation from particular RAS or from particular ecosystems, without associated species names. Sauder et al. (2011) used rtPCR to look at *amoA* and *16S* rRNA genes of nitrifiers in the biofilters of freshwater and saltwater hobby-scale aquaria, i.e., smaller systems with lesser waste loadings than commercial aquaculture RAS. Saltwater system biofilters showed both AOB and AOA, the latter dominating in five of eight systems, and the authors suggested that both communities contribute substantially to nitrification. The 84 archaeal *amoA* clones included Candidatus *N. maritimus* and Candidatus *Cenarchaeum symbiosum* A. Sakami et al. (2012) assayed *amoA* DNA sequences in three biofilters for marine RAS producing unspecified fish under various environmental conditions. Among the AOBs were 19 OTUs, most not affiliated with a species. Those identified included four *Nitrospira*, four *Nitrosomonas*, and one *Nitrosococcus* species or strains. In contrast, the AOBs in the RAS system that we characterized were dominated by a wider range of *Nitrosomonas* species and strains. Among 240 archaeal *amoA* sequences examined by Sakami et al. (2012) were 63 distinct clones. Most were not identified to species, *N. maritimus* being the only one identified. Our results showed two strains of that species, three named and five unnamed congeners, and six archaeal OTUs of unknown affiliation. Analyzing the biofilter nitrifier community in a marine RAS producing unspecified shrimp, Brown et al. (2013) examined AOB populations by targeting *16S* rRNA and AOA by using *amoA* genes. Archaeal *amoA* genes were more abundant in all compartments of the RAS than bacterial *amoA* genes. In results similar to ours, most AOB were related to *Nitrosomonas marina* and most AOA to *N. maritimus*. Using DGGE to characterize thaumarchaeal *16S* rRNA genes, Bagchi et al. (2014) also showed low AOA diversity. Thus, there are considerable differences among studies, even those using similar methods. These differences may be attributable to the environmental conditions (see following sections), systems operations conditions (Bartelme et al. 2017), or source of seeding (Gross et al. 2003).

Environmental factors, such as pH, oxygen concentration and temperature, will affect the compositions of microbial communities responsible for nitrification. Both AOB and AOA are sensitive to a number of environmental conditions (reviewed by You et al. 2009); the dynamics of nitrifying organisms in response to nutrient removal and water quality remains poorly understood. Hence, it is necessary to explore and better understand how AOB and AOA respond to environmental perturbations and affect water quality in a diversity of aquaculture systems.

### Ammonia oxidizing bacterial community composition

Ammonia-oxidizing bacteria are distinguished on the basis of DNA homology, GC content of DNA, cellular shape and ultrastructure, as well as characters relevant to environmental adaptation, such salt requirement, ammonia tolerance, ability to utilize urea, and whole-cell proteomics (Koops et al. 1991). Using a metagenomics approach, we characterized the AOB community on the biofilters of a marine aquaculture facility producing hybrid grouper and uncovered a diversity of both described and undescribed species. Those for which environmental adaptations are known exhibited contrasting environmental optima. We observed three *amoA* gene sequences with high homology to three different strains of *N. europaea*, a microbe first isolated from soil and common in soil, freshwater, and sewage, with no requirement for salt (Koops et al. 1991). *N. europaea* needs high concentrations of ammonia for energy to grow and divide, and hence grows slowly; it tolerates a pH of 6.0–9.0, with slightly basic conditions being optimal; it has aerobic metabolism, and prefers temperatures between 20 and 30 °C (Chain et al. 2003). We observed one *amoA* sequence from *N. stercoris*, which occurs in strongly eutrophic environments such as wastewater treatment plants; that they grow at 1000 mM ammonium suggests that these ammonia oxidizers contribute to removing high concentrations of ammonia under highly eutrophic conditions (Nakagawa and Takahashi 2015). One *amoA* sequence was from *N. cryotolerans*, a cold-tolerant obligate halophilic bacterium first isolated from Alaskan marine waters (Jones et al. 1988). We had one observation of *N. eutropha*, a bacterium found in highly eutrophic environments such as sewage disposal systems; it tolerates elevated ammonia concentrations, can grow anaerobically, and uses nitrite as an electron acceptor and hydrogen as a reductant. We observed two *amoA* sequences from *N. aestuarii*, which is common in marine and estuarine waters and requires salt, with optimum growth around 17.5 ppt NaCl (Koops et al. 1991). We observed eight distinct *amoA* sequences from different strains of *N. marina*, which occurs in marine waters and salt lakes; it requires salt and exhibits optimum growth at around 20 ppt NaCl (Koops et al. 1991).Observation of a diverse assemblage of *Nitrosomonas* and perhaps other ammonia-oxidizing bacteria with differing optimal ammonia and salinity concentrations, as well as differing requirements for oxygen and electron receptors, suggests that ammonia oxidation can go forward under a range of environmental conditions. This diversity also suggests that ammonia will be oxidized effectively in different microhabitats within biofilms throughout the treatment train. These inferences suggest how the process of ammonia oxidation proved resilient in the face of changing environmental conditions through time for fish production within the RAS that we characterized, especially regarding heightened ammonia loadings as fish density increased through the production cycle.

### Ammonia oxidizing archaeal community composition

Growing understanding of physiological differences among AOA suggests adaptive differentiation, which may support niche partitioning in the natural environment. Ergruder et al. (2009) outlined the possible niches of AOA, and proposed that the AOA might be important actors within the nitrogen cycle in low-nutrient, low-pH, and sulfide-containing environments. We suggest the possibility of niche partitioning within aquaculture biofilters, which in turn suggests heightened biofilter function and resiliency, as follows. The majority of AOA in marine ecosystems belong to Group 1.1a or Marine Group I within the candidate order *Nitrosopumilales* (Stieglmeier et al. 2014), which includes *N. maritimus* SCM1 (Konneke et al. 2005). Archaea closely related to *N. maritimus* SCM1 subsequently were isolated from marine and estuarine sediments (Mosier et al. 2012; Park et al. 2014) and coastal waters (Qin et al. 2014). Although all representatives of Group 1.1a couple ammonia oxidation with autotrophic carbon fixation, they vary in metabolic traits including the ability to utilize urea and dependence on small amounts of organic compounds (Park et al. 2014; Qin et al. 2014). Archaea of genus *Nitosopumilis* can use ammonia as an energy source and perform carbon fixation through the 3-hydroxypropionate/4-hydroxybutyrate pathway (Berg et al. 2007). Physiological and genomic data show that each strain has different metabolic and functional traits that may reflect contrasting modes of life (Bayer et al. 2016). Gene sequences similar to those of *N. maritimus* have been PCR-amplified from pelagic ocean waters (Karner et al. 2001; Wuchter et al. 2006) and a wastewater treatment plant (Park et al. 2006). *N. koreensis* was first isolated from sediment collected from the Arctic Ocean (Park et al. 2010). Genes for multicopper oxidase and blue copper domain-containing proteins that are potentially involved in energy conservation through ammonia oxidation were less enriched in the genome of *N. koreensis* strain AR1 than in other members of the genus (Park et al. 2012). Absence of a hydroxylamine oxidoreductase gene in strain AR1 indicated a novel ammonia oxidation pathway. While the genome contained genes for the 3-hydroxypropionate/4-hydroxybutyrate pathway of carbon fixation, the high frequency (~ 30%) of unique genes in strain AR1 suggested the potential for niche differentiation among sediment-dwelling AOA. For example, The AR1 genome lacked a high-affinity phosphate-uptake operon found in the genome of *N. maritimus*. *Candidatus N. piranensis* strain D3C and *Candidatus N. adriaticus* strain NF5 were obtained from the same location and are closely related phylogenetically, but appeared to exhibit different metabolic traits and functional adaptations (Bayer et al. 2016). *N. piranensis* D3C is non-motile, shows versatility in substrate utilization, and can use urea as an alternative substrate to ammonia. It has a second, divergent copy of the *AmoB* subunit of ammonia monooxygenase, which suggests additional catalytic function and greater metabolic versatility. *N. adriaticus* NF5 contains many chemotaxis-related genes, can express archaella (whip-like structures for motility), and may sense and actively seek favorable microenvironments such as nutrient-rich particles (Bayer et al. 2016). Factors influencing the diversity of AOA in biofiltration systems remain unknown and largely unexplored; Urakawa et al. (2008) suggested that salinity, ammonium concentration, pH, and temperature may be the most important factors. We hypothesize that contrasting environmental optima among *Nitrosopumilus* species and archaea generally might contribute to their coexistence in biofilters, and that the presence of a consortium of such species may contribute to heightened nitrification and functional resiliency. This hypothesis might be tested by collecting and characterizing biofilm from different points in the treatment train and through the production cycle as ammonia loading increases.

### Are ammonia-oxidizing archaea important in marine aquaculture biofilters?

Ammonia oxidation long was thought to be performed mainly by certain lineages of *β*- and γ-*Proteobacteria* (Purkhold et al. 2000). However, the discovery of ammonia-oxidizing archaea (Konneke et al. 2005; Treusch et al. 2005) changed perception of microbial nitrification and nitrogen cycling and led to questions regarding nitrifier diversity, the contribution of archaea, and the ecology and evolutionary origins of ammonia oxidation-based metabolism. The *Crenarchaeota* taxon to which *Notrosopumilis* belongs is estimated to comprise 20–30% of ocean picoplankton (Karner et al. 2001). Metagenomic and biomarker data have shown nitrification by Group 1 *Crenarchaeota* in a variety of mesophilic aerobic environments. In the North Sea, the ammonia oxidation rate corresponded to numbers of cells and *amoA* copies of *Crenarchaea* species exhibiting close sequence identity to *N. maritimus,* while bacterial *amoA* copies remained 1–2 orders of magnitude less common (Wuchter et al. 2006). While per-cell rates of ammonia oxidation are less for AOA (2–4 × 10–15 mol NH_3_/cell/day, Wuchter et al. 2006) than for AOB (6–20 × 10–15 mol NH_3_/cell/day, Ward 1987), observation of 1000-3000-times greater abundance of AOA than AOB in marine environments (Karner et al. 2001; Wuchter et al. 2006) suggested that archaea may be the more important nitrifiers. The ammonia affinity and oxidation kinetics exhibited by *N. maritimus* strain SCM1 under oligotrophic conditions adapts it to life under conditions of nutrient limitation (Martens-Habbena et al. 2009); the threshold concentration needed for growth of strain SCM1 could be as low as 10–20 nM ammonium, which suggests that *Nitrosopumilus*-like AOA could compete successfully for nitrogen with heterotrophic bacterioplankton and phytoplankton.

Using PCR primers targeting archaeal amoA, Francis et al. (2005) found AOA to be pervasive in areas of the ocean that are critical for the global nitrogen cycle, including the base of the euphotic zone, suboxic water columns, and estuarine and coastal sediments. Diverse and distinct AOA communities were associated with each of these habitats, with little overlap between water columns and sediments. Within marine sediments, most AOA sequences are unique to individual sampling locations, whereas a small number of sequences are evidently cosmopolitan in distribution. Considering the abundance of non-extremophilic archaea in the ocean, their results suggest that AOA may play a significant, but previously unrecognized, role in the global nitrogen cycle. Walker et al. (2010) reported the 1,645,259-bp genome of Candidatus *N. maritimus* strain SCM1, revealing highly copper-dependent systems for ammonia oxidation and electron transport that are distinctly different from known ammonia-oxidizing bacteria. The conservation of *N. maritimus* genome organization and gene content among marine metagenomes indicates that the unique physiology of these ammonia-oxidizing archaea may play a significant role in the nitrogen cycle. The contribution of AOA to nitrification in RAS biofilters, however, is yet unresolved. Given the high affinity of *Nitrosopumilus*-like AOA and its reaction kinetics, the reasonably high diversities of AOA that we and Urakawa et al. (2008) observed would suggest considerable ammonium flux through this component of the biofilm community. However, the low abundance of Crenarchaeotan archaea in a trickling filter biofilm observed by Foesel et al. (2008) using fluorescent in situ hybridization led them to assess their contribution to overall nitrification to be negligible. RAS biofilters host complex microbial communities with multiple ammonia oxidation pathways and whose composition is affected by system operations (Bartelme et al. 2017). Examining the temporal and spatial stability of AOB and AOA in biofilters, Bagchi et al. (2014) used qPCR to quantify *amoA* gene abundance and showed that in marine systems, AOB outnumbered AOA by three to five orders of magnitude. Using *16S* rRNA and *amoA* barcoding, Bartelme et al. (2017) showed that *Nitrosomonas* was present at all water depths and sampling times, but their abundance was three orders of magnitude less than that for AOA and varied over time.

That our results differed in some regards from those of some other studies supports the view that AOB and AOA communities vary widely with culture and environmental conditions, particularly substrate availability, and perhaps also with the environmental conditions under which the culture water was taken (Sakami et al. 2012). The relative abundance of AOB and AOA and their contributions to nitrification may vary with environmental conditions, in particular, with substrate availability. Under ammonia-limited conditions, organisms with *Nitrosopumilus*-like kinetics may outcompete AOB and dominate nitrification, while AOB may be more competitive in environments higher nutrient levels. Organic matter-rich particles may provide niches for nitrifiers with significantly different kinetic properties (Karl et al. 1984; Phillips et al. 1999; Martens-Habbena et al. 2009), such as different members of the *Nitrosomonas eutropha* lineage (Phillips et al. 1999). We note that organic-rich particles are abundant in RAS, and their presence may affect the rate of nitrification and the microbial subcommunity responsible for it. Brown et al. (2013) noted that water quality and biofilm attachment media played a role in the competitiveness of AOA over AOB and among different species of *Nitrospira*. Using qPCR, Sauder et al. (2011) detected both thaumarchaeal and bacterial *amoA* genes in all saltwater samples they examined, with AOA genes outnumbering AOB genes in five of eight biofilters. The compositions of ammonia-oxidizing communities in freshwater and marine systems were distinct, and composite clone libraries of AOA *amoA* genes revealed distinct freshwater and saltwater clusters. Sequencing 16S rRNA amplicons, Gonzalez-Silva et al. (2016) showed significantly different AOB communities in cultures originating from freshwater, brackish (20‰) and seawater environments; 60% of the total operational taxonomic units (OTUs) in the ammonia-oxidizing bacteria (AOB) were unique to that environment. Real-time PCR would provide an assessment of the level of *amoA* gene expression, while assessment of ammonia flux (Ward 1987; Wuchter et al. 2006) would provide the direct measure of greatest interest. More thorough understanding of the ammonia-oxidizing community may promote more targeted seeding to initiate nitrification in RAS biofiters (Gross et al. 2003) and to support management of biofilters through the production cycle.

## Abbreviations

amoA: ammonia monooxygenase gene
AOA: ammonia-oxidizing archaea
AOB: ammonia-oxidizing bacteria
COD: chemical oxygen demand
DGGE: denaturing gradient gel electrophoresis
FCR: feed conversion ratio
NOB: nitrite-oxidizing bacteria
OTU: operational taxonomic unit
PCR: polymerase chain reaction
RAS: recirculating aquaculture system
TAN: total ammonia nitrogen
UV: ultraviolet
16S rRNA: 16S ribosomal RNA gene
